# Safety and efficacy of Galgeun-tang-ga-cheongung-sinyi, a herbal formula, for the treatment of chronic rhinosinusitis: A study protocol for a randomized controlled trial

**DOI:** 10.1097/MD.0000000000011196

**Published:** 2018-06-22

**Authors:** Mi Ju Son, Ojin Kwon, Sungha Kim, Young-Eun Kim, So Young Jung, Bo-Young Kim, Jeong In Kang, Jun-Hwan Lee, Dong-Hyo Lee

**Affiliations:** a Korea Institute of Oriental Medicine; b Korean Medicine Life Science, University of Science & Technology, Daejeon; c Department of Ophthalmology & Otolaryngology & Dermatology, Woo-Suk University Korean Medicine Hospital, Jeonju-si, Jeollabuk-do, Republic of Korea.

**Keywords:** chronic rhinosinusitis, herbal formula, Galgeun-tang-ga-cheongung-sinyi, Gegen-tang-jia-chuanxiong-xinyi, Kakkontokasenkyushin’i, placebo, randomized controlled trial

## Abstract

**Introduction:**

A herbal formula, Galgeun-tang-ga-cheongung-sinyi (GGTCS), is traditionally used for the treatment of rhinosinusitis in East Asian countries. However, there is a dearth of clinical evidence supporting the effects of this medication. Here, we describe the protocol for a randomized controlled study designed to investigate the efficacy and safety of GGTCS for the treatment of chronic rhinosinusitis (CRS).

**Methods and analysis:**

To investigate the clinical efficacy and safety of GGTCS for the treatment of CRS, a randomized, double-blind, placebo-controlled, parallel group, clinical trial has been designed. A total of 58 participants with CRS will be recruited and randomly allocated to a GGTCS or placebo group in a 1:1 ratio. The participants will be administered GGTCS or placebo granules 3 times a day for 8 weeks. Data will be collected from the participants at baseline and at 1, 2, 4, and 8 weeks after random allocation. The primary outcome measure will be the mean change in the Sino-Nasal Outcome Test-22 score from baseline to 8 weeks. The secondary outcomes will include the Total Nasal Symptom Score, EuroQoL 5 Dimensions 5 Levels score, Nasal Endoscopy Index, Lund-Mackay score, and total serum immunoglobulin E level.

**Discussion:**

The key elements for conducting a high-quality randomized clinical trial have been addressed in this protocol. In summary, the findings of this study are expected to provide a base for large-scale randomized controlled trials to confirm the safety and efficacy of GGTCS for the treatment of CRS and may consequently serve to improve future treatment strategies for this condition.

**Trial registration:** This study has been registered at the Korean National Clinical Trial Registry, Clinical Research Information Service (KCT0002835).

## 1 Introduction

Chronic rhinosinusitis (CRS) is defined as a disease characterized by inflammation of the nasal and paranasal sinus mucosa that persists for more than 12 weeks.^[1]^ CRS is one of the most common chronic diseases, affecting 11.8% to 17.4% of the American population^[2]^ and 10.9% of the European population.^[3]^ This condition not only causes physical problems such as nasal congestion, thick mucus production, and loss of olfaction, but also impacts psychological wellbeing and daily functioning.^[4]^ Indeed, CRS is associated with a lifetime of medical and surgical resource consumption, resulting in significant health care expenditures; the range for overall CRS-related health care costs has been reported as $6.9 to $9.9 billion USD per year.^[5]^

CRS is a multifactorial disease with an elusive pathology; therefore, there are no specific targets for therapeutic interventions. Considering that it is an inflammatory disease, anti-inflammatory therapy including corticosteroids and antibiotics plays a role in its treatment. Although corticosteroids are known to be effective against nasal polyps, they can increase the risk of epistaxis.^[6,7]^ Moreover, antibiotics show limited treatment effects and are associated with gastrointestinal disturbances and allergic reactions such as skin irritation.^[8]^ In cases of marked mechanical obstruction of the airways or chronic disease that is unresponsive to maximal medical therapy, surgical intervention is the treatment of choice, although its effects are inconclusive.^[9]^

Because of the limited success of conventional therapy and the nature of the condition, herbal medicines are becoming increasingly popular and are frequently used by patients with rhinosinusitis in East Asian countries.^[10,11]^*Galgeun-tang* (GGT) is a herbal formula composed of 7 medicinal herbs (*Puerariae Radix*, *Ephedrae Herba*, *Cinnamomi Ramulus*, *Paeoniae Radix*, *Glycyrrhizae Radix et Rhizoma*, *Zingiberis Rhizoma Recens*, and *Zizyphi Fructus*). It has anti-inflammatory and immunoregulatory properties.^[12,13]^*Galgeun-tang-ga-cheongung-sinyi* (GGTCS, *Gegen-tang-jia-chuanxiong-xinyi* in Chinese, *Kakkon-to-ka-senkyu-shin’i* in Japanese) is a herbal prescription that is prepared by the addition of *Cnidii Rhizoma* and *Magnoliae Flos* to GGT and is also known to have anti-inflammatory effects.^[14]^ It is considered effective for otorhinolaryngological diseases^[15]^ and is used for the treatment of rhinosinusitis throughout East Asia.^[16]^

Despite the clinical experiences and experimental results supporting the use of GGTCS,^[14,15]^ few clinical trials have evaluated the effects of GGTCS for CRS. Here, we describe the protocol for a randomized controlled trial designed to assess the efficacy and safety of GGTCS for the treatment of CRS compared with placebo. This trial has been designed to better reflect the characteristics of traditional medicine by considering the traditional constitutional concept of GGTCS.

## 2 Methods

### 2.1 Objective

The aim of this study was to describe the protocol for a randomized controlled trial designed to clinically assess the efficacy and safety of GGTCS for the treatment of CRS.

### 2.2 Study design and setting

**Figure 1:**
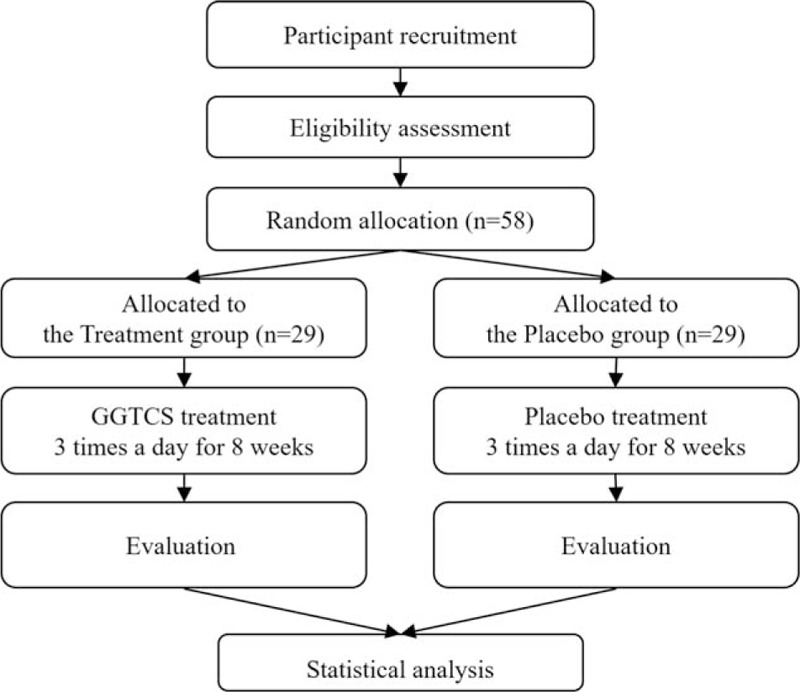
Flowchart describing the study plan. GGTCS = Galgeun-tang-ga-cheongung-sinyi.

This randomized, double-blind (patient, practitioner, and assessor), placebo-controlled, clinical trial will be conducted at the Woosuk Korean Medicine Medical Center in the Republic of Korea. The study period will include 8 weeks of medication and follow-up. The design is summarized in Fig. 1 and Table 1. The study protocol (version 1.1, 11 April 2018) follows the Standard Protocol Items: Recommendations for Interventional Trials (SPIRIT) guidelines (see Additional File 1).

**Table 1:**
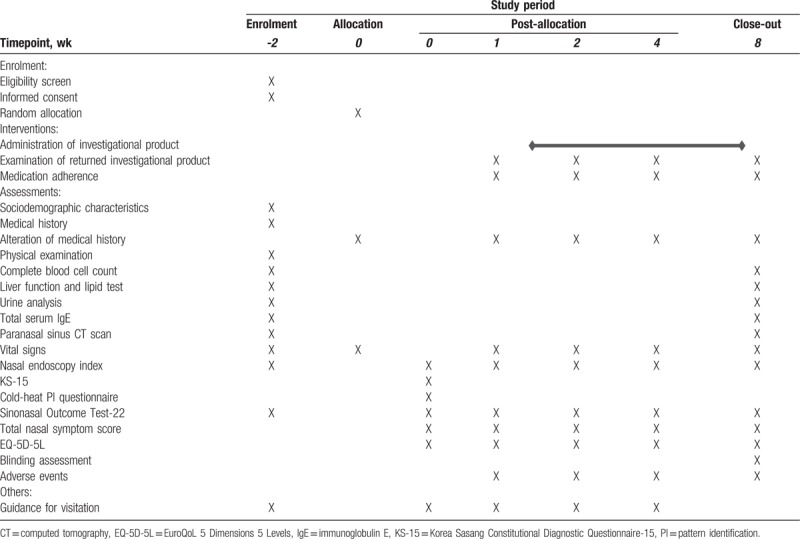
Schedule of enrolment, interventions, and outcome measurements for a randomized controlled trial assessing the safety and efficacy of Galgeun-tang-ga-cheongung-sinyi, a herbal formula, for the treatment of chronic rhinosinusitis.

### 2.3 Recruitment

Participant recruitment will begin in April 2018 at the Woosuk Korean Medicine Medical Center, and it is expected to be completed in December 2018. The trial will be advertised in local newspapers, public transportation, and the hospital, and a total of 58 participants will be recruited. Written informed consent will be obtained from all study participants before enrolment, and participants may decline to participate or withdraw at any time without disadvantage. The written informed consent form will include information on the background and purpose of the study, experimental and placebo drugs, outcome measures, and possible benefits and harms. The participants will be immediately notified when new facts regarding the study are found.

### 2.4 Participant inclusion and exclusion criteria

#### 2.4.1 Inclusion criteria

Participants will be considered eligible for inclusion if they meet the following criteria:

(1) Men or women aged 19 to 60 years at the screening visit;
(2) A diagnosis of CRS based on the presence of rhinosinusitis symptoms for more than 12 weeks.
  A. Presence of more than 2 major symptoms or 1 major symptom with 2 minor symptoms:
    i. Major symptoms: facial pain/pressure, nasal obstruction/blockage, nasal discharge/purulence/discolored postnasal drainage, hyposmia/anosmia, purulence observed in the nasal cavity on examination
    ii. Minor symptoms: headache, fever, halitosis, fatigue, dental pain, cough, ear pain/pressure/fullness
  B. A diagnosis of rhinosinusitis based on paranasal sinus computed tomography (CT) findings.
(3) A Sino-Nasal Outcome Test-22 (SNOT-22) score of ≥ 20 at the screening visit
(4) Ability to comprehend the purpose and process of the study as well as the properties of the investigational drug and voluntary provision of written informed consent

#### 2.4.2 Exclusion criteria

The exclusion criteria will be as follows:

(1) A previous history of nose-related or allergy-related diseases and treatment
  A. Nasal polyps, cystic fibrosis, primary ciliary dyskinesia, untreated deviated nasal septum
  B. Acute bacterial exacerbation of CRS (acute pain, acute pressure, fever, pus on discharge)
  C. Acute complication of CRS (abscess)
  D. Orbital or central nervous system complications of CRS
  E. Acute respiratory infection developed within the last 7 days
  F. Use of topical decongestants or cromolyn sodium within the last 3 days
  G. Use of antibiotics, antihistaminics, anticholinergics, and intranasal steroids within the last 1 week
  H. Use of oral steroids or leukotriene receptor antagonists within the last 4 weeks
  I. Receipt of immunotherapy within the last 5 years
(2) Presence of serious medical conditions that could interfere with clinical trial participation
  A. Uncontrolled hypertension (systolic blood pressure ≥ 160 mm Hg and diastolic blood pressure ≥ 100 mm Hg);
  B. Uncontrolled diabetes mellitus (HbA1c ≥ 6.5%);
  C. Abnormal kidney function test results showing blood creatinine levels that are more than twice the upper limit of the normal range;
  D. Abnormal liver function test results showing alanine aminotransferase or aspartate aminotransferase levels that are more than twice the upper limit of the normal range;
  E. Serious hyperlipidemia, anemia, active tuberculosis, thyroid disease, and other serious inflammatory and systemic diseases;
  F. Past or present history of malignant tumors.
(3) Pregnancy, planning a pregnancy, or breastfeeding
(4) Hypersensitivity to the investigational drug or any of its components
(5) Participation in other clinical trials within the past 1 month
(6) Communication difficulties that will not allow proper compliance with the investigator's instructions
(7) Ineligibility for participation as judged by the investigator

At any time during the clinical trial, the participant may voluntarily withdraw from the clinical trial or also be discontinued at the researcher's discretion. If at any time during the clinical trial period, an adverse event (AE) determined to be harmful to the participant is observed, the researcher may temporarily discontinue the treatment of that participant. If continuing the trial is harmful to the participant based on the progression of the AE and its causal relationship with the drug, then the researcher will permanently discontinue the clinical treatment of that participant. Anyone who is discontinued early from the study due to an AE may receive the appropriate treatment for the AE, when necessary. Anyone who is discontinued early from the study owing to an AE must be assessed continuously by the researcher or someone designated by the researcher until the AE is resolved or is determined to be permanent.

### 2.5 Intervention

Participants will be randomly assigned to the GGTCS or placebo control group in a ratio of 1:1. They will receive treatment or evaluation according to the predetermined schedule. All participants will orally receive GGTCS or placebo 3 times a day 30 minutes before breakfast, lunch, and dinner or between meals for 8 weeks. The drugs for this clinical trial will be provided to the participants at baseline and at 1, 2, and 4 weeks after baseline. To confirm adherence to the medication regimen, participants will be requested to return unused drugs as well as spent wrappers of used drugs.

GGTCS consists of *Puerariae Radix*, *Ephedrae Herba*, *Cinnamomi Ramulus*, *Paeoniae Radix*, *Glycyrrhizae Radix et Rhizoma*, *Zingiberis Rhizoma Recens*, *Zizyphi Fructus*, *Cnidii Rhizoma*, and *Magnoliae Flos*. All constituents of this formulation comply with Korean Pharmacopoeia standards. The placebo will comprise corn starch, lactose hydrate, citric acid hydrate, ginseng flavored powder, and caramel coloring. Both drugs will be identical in appearance and color (brown) and will be packaged in identical white, opaque wrappers. The detailed components of GGTCS are described in Table 2. Both GGTCS and the placebo are manufactured by Hanpoong Pharm. Co., Ltd. (Wanju, Republic of Korea) according to good manufacturing practice standards. Quality control and quality assurance for quality and safety testing, packaging, and contents of the treatment drug, including checks for potential contaminants such as heavy metals or steroids, will be undertaken by the manufacturers to ensure drug stability and quality.

**Table 2:**
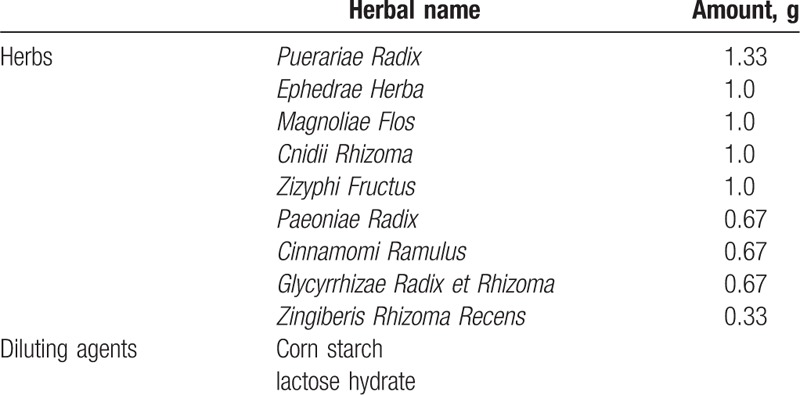
Composition of the herbal formula Galgeun-tang-ga-cheongung-sinyi.

In principle, participants of both groups will be prohibited from concomitant use of any prescription and nonprescription medicines, functional health foods, or herbal medicines that can potentially affect the outcome of this experiment until after the follow-up period of 8 weeks. Participants who have been receiving medication for chronic diseases before initiation of this study will be allowed to continue their regimen, and care will be taken not to alter the dosage or type of drug.

### 2.6 Outcomes

The time points for the outcome measurements are presented in detail in Table 1.

#### 2.6.1 Primary outcome

The primary outcome will be the mean change in the SNOT-22 score from baseline to 8 weeks.^[17]^ The SNOT-22 is a validated, rhinosinusitis-specific, quality-of-life instrument comprising 22 items encompassing 4 subscales: nasal symptoms, ear and facial symptoms, sleep condition, and psychological domains.^[18]^ The participants will be asked to score a list of 22 symptoms and mark the 5 most important items.

#### 2.6.2 Secondary outcomes

The secondary outcomes will include the mean change in the Total Nasal Symptom Score (TNSS), EuroQoL 5 Dimensions 5 Levels (EQ-5D-5L) score, Nasal Endoscopy Index, Lund-Mackay score, and total serum immunoglobulin E (IgE) level. The TNSS assesses 4 nasal symptoms (nasal obstruction, rhinorrhea, itching, and sneezing) on a 4-point scale: 0, no symptoms; 1, mild symptoms; 2, moderate symptoms; and 3, severe symptoms.^[19]^ The EQ-5D-5L questionnaire is brief, consisting of 5 questions and a 5-point scale that documents the patient's overall health status.^[20]^ The Nasal Endoscopy Index was developed and validated for the assessment of nasal cavity conditions from 4 aspects: color, edema or atrophy, dryness or dampness, and the amount and characteristics of rhinorrhea. The patients’ nasal cavities will be examined with a nasal endoscope at every visit. Photos of the anterior nasal cavity will be obtained using the KAU-3000 HARMONY ENT treatment unit (KASAMA ENT Co, Ltd., Incheon, Republic of Korea), and the acquired photos will be scored according to assessment guidelines for the Nasal Endoscopy Index.^[21]^ The Lund–Mackay score is a widely used method for radiological staging of CRS.^[22]^ CT images of the paranasal sinuses and ostiomeatal complex will be scored from 0 to 2 (0, no abnormality; 1, partial opacification; and 2, complete opacification) by an independent radiology specialist. We will also examine the total serum IgE level to evaluate the immune-regulatory effects of GGTCS.

#### 2.6.3 Other measures

Participants will also be asked to complete the cold-heat pattern identification questionnaire and Korea Sasang Constitutional Diagnostic Questionnaire-15 (KS-15) at the first visit in order to identify the correlation between the efficacy of GGTCS, the cold-heat pattern, and the Sasang constitutional type.

### 2.7 Randomization and allocation concealment

A statistician will generate random allocation numbers using a computer program [Strategic Applications Software (SAS), version 9.4; SAS Institute Inc., Cary, NC]. The generated numbers will be sealed in opaque envelopes and stored in double-locked cabinets. Participants who fulfil all inclusion criteria will be assigned to 1 of the 2 groups via blocked randomization.

### 2.8 Blinding

The participants, investigators, coordinators, pharmacist, monitoring agent, and statistician will be blinded to the group allocation data, which will be known only to the person in charge of random allocation. The statistician will create a random allocation table indicating assignment to group A or B. The person in charge of random allocation will deliver this allocation table and group information to the pharmaceutical company (because there is no resource to create a randomization list at the investigational products manufacturing company, our team will prepare the randomization table). The pharmaceutical company will make and label the investigational products on the basis of this information. The label will include each participant's identification number (R1001 to R1058; identical to the random allocation numbers) and visit number (V1, V2, V3, and V4). An opaque emergency envelope containing allocation information will be prepared and stored in a safe place in anticipation of unexpected events. Violation of blinding will be considered only under circumstances where knowledge of the medication being administered to a participant is essential for treatment. The validity of blinding will be assessed according to the new blinding index.

### 2.9 Sample size

No clinical trial has been conducted to evaluate the effects of GGTCS on the SNOT-22 score for patients with CRS. A formal power calculation has not been performed because this study will evaluate the feasibility to calculate a required sample size for subsequent definitive randomized clinical trials. On the basis of recommendations by a previous study,^[23]^ a minimal effect size of 0.25 and dropout rate of 20% have been assumed for this trial. Thus, a total of 58 participants, 29 in each group, will be recruited.

### 2.10 Data and safety monitoring

Regular monitoring will be conducted to ensure quality control of the data according to the planned protocol and standard operating procedures. Data quality will be ensured by regular monitoring. The monitoring agent will confirm whether the data are consistent with the source documents and whether the trial is conducted according to the approved protocol. All AEs observed during the study period will be recorded and reported. In case severe AEs and crucial issues occur, the investigators will determine whether these issues are acceptable and whether the trial should be amended or ended. The trial data will be saved in an electronic data capture system (Medidata Rave; Medidata Solutions Inc., New York, NY).

### 2.11 Safety and adverse events

For the safety of the study participants, complete blood cell counts, liver function tests, lipid tests, and urine analyses will be performed during the screening phase and at the end of the trial. Vital signs will be examined at every visit. Contact information for AE reporting at any time will be provided to all participants.

AEs associated with GGTCS have not yet been revealed. However, it is assumed that pseudoaldosteronism, myopathy, rash, pruritus, anorexia, gastric discomfort, nausea, diarrhea, autonomic dysfunction, and voiding dysfunction can occur in association with the individual herbal components of GGTCS. Any AE reported by participants will be recorded in the “Adverse Event Record Table.” A causal relationship between the event and GGTCS or placebo treatment will be evaluated using a 6-point scale: 1, definitely related; 2, probably related; 3, possibly related; 4, probably not related; 5, definitely not related; and 6, unknown. The seriousness of AEs will be scored using a 3-point scale: 1, mild; 2, moderate; and 3, severe. Any AEs will be reported in accordance with the regulations of the institutional review board (IRB).

### 2.12 Statistical analysis

The measured variables, including the primary and secondary outcomes, will be assessed using the full analysis set based on intention-to-treat principles. The per-protocol analysis set will be used for the sensitivity analysis. Values for baseline characteristics in both groups will be expressed as means and standard deviations for continuous variables satisfying the normal assumption or medians and interquartile ranges for non-normal data. Frequencies and percentages will be used to represent categorical variables. Baseline differences between groups will be assessed using an independent 2-sample *t* test or the Wilcoxon rank-sum test for continuous variables and the Chi-square test or Fisher exact test for categorical variables.

A mixed-effect model repeated measure (MMRM), which sets participants as a random factor and the group and visit as fixed factors, will be performed to analyze between-group differences in the primary and secondary outcome measures. A subgroup analysis will also be performed to explore differences in the effects of GGTCS according to the cold-heat pattern, Sasang constitutional type, and symptom severity. Student paired *t* tests or Wilcoxon signed-rank tests will be performed to analyze differences before and after treatment in each group, and repeated measures analysis of variance will be used to assess differences between groups at each visit. The level of significance will be set at 0.05 (2-tailed), and all analyses will be performed by an independent statistician using SAS version 9.4 (SAS institute. Inc., Cary, NC).

## 3 Discussion

GGTCS is an herbal formula that has been approved by the Ministry of Food and Drug Safety (MFDS) of the Republic of Korea, where it is commercially marketed for nasal congestion, sinusitis, and chronic rhinitis.^[24]^ Numerous herbal medicines, including GGTCS, are recognized as components of traditional medicine treatment, and have been approved by the MFDS based on the historical medical literature and long-term experience of use. Thus, clinical evidence is lacking for several herbal medicines. The study outlined in this protocol aims to explore the efficacy and safety of GGTCS for the treatment of CRS in the clinical field. We may draw supportive evidence for the use of GGTCS through this study.

CRS is classified as CRS without nasal polyps (CRSsNP) and CRS with nasal polyps (CRSwNP). CRSsNP seems to mechanistically involve Th1 mucosal inflammation,^[25]^ whereas CRSwNP seems to be associated with Th2 skewing.^[26,27]^ It is being increasingly recommended that treatment decisions should be made on the basis of an understanding of the patient's CRS phenotype and likely etiology.^[28]^ Because we need to include a phenotype that reflects the latest therapeutic trends, we plan to recruit patients with CRSsNP for the present study. This will also be more feasible because the prevalence of CRSsNP is higher than that of CRSwNP^[29]^; moreover, GGTCS is usually prescribed to patients with CRSsNP in Korean Medicine clinics. However, our findings will not preclude the efficacy of GGTCS for the treatment of CRSwNP, as there is evidence that GGT and its components have anti-allergenic as well as anti-inflammatory effects.^[30,31]^ Further studies for the efficacy and safety of GGTCS for the treatment of CRSwNP will be designed and conducted.

GGT is usually prescribed to Tae-eum type patients classified according to the Sasang constitutional medicine (SCM) theory, which is a Korean traditional medicine theory that categorizes humans into 4 distinct constitutions: Tae-yang, Tae-eum, So-yang, and So-eum. According to this theory, each type shows different sensitivities to some types of herbs and medicines.^[32]^*Puerariae Radix*, *Ephedrae Herba,* which are main ingredients of GGTCS, are known as medicinal herb for Tae-eum type patients. Assuming that the type and frequency of AEs caused by GGTCS for CRS treatment will differ among SCM types, we will use the KS-15 questionnaire in the present study to derive results that can serve a reference for planning future large-scale clinical trials reflecting the characteristics of traditional medicine.

The key elements for conducting a high-quality randomized clinical trial have been addressed in this protocol. In summary, the findings of this study are expected to provide a base for large-scale randomized controlled trials to confirm the safety and efficacy of GGTCS for the treatment of CRS and may consequently serve to improve future treatment strategies for this condition.

## Author contributions

MJS conceived and designed the study protocol and drafted the manuscript. YEK and SK helped in drafting the manuscript. OK, SYJ, BYK, and JIK made substantial contributions to the protocol design. JHL and DHL made the final decision to submit for publication. All authors have read and approved the final manuscript for submission.

**Conceptualization:** Mi Ju Son.

**Funding acquisition:** Jun-Hwan Lee.

**Investigation:** Mi Ju Son, So Young Jung, Bo-Young Kim, Jeong In Kang, Dong-Hyo Lee.

**Methodology:** Ojin Kwon, Sungha Kim, So Young Jung, Bo-Young Kim, Jeong In Kang.

**Software:** Ojin Kwon.

**Supervision:** Dong-Hyo Lee.

**Writing – original draft:** Mi Ju Son.

**Writing – review & editing:** Sungha Kim, Young-Eun Kim, Dong-Hyo Lee.
